# Superior mesenteric artery syndrome coexists with Nutcracker syndrome in a female: a case report

**DOI:** 10.1186/s12876-019-0932-1

**Published:** 2019-01-23

**Authors:** Ying Shi, Guangya Shi, Zhenyu Li, Yanfang Chen, Shaohui Tang, Wei Huang

**Affiliations:** 1 Department of Gastroenterology, The First Affiliated Hospital, Jinan University, Guangzhou, 510630 People’s Republic of China; 2 The First Clinical Medical College, Jinan University, Guangzhou, 510630 People’s Republic of China; 3 Department of Gastroenterology, The People’s Hospital of Fujian Province, 350004 Fujian, People’s Republic of China; 4 Department of Radiology, The First Affiliated Hospital, Jinan University, Guangzhou, 510630 People’s Republic of China

**Keywords:** Superior mesenteric artery, Nutcracker syndrome

## Abstract

**Background:**

Superior mesenteric artery (SMA) syndrome, also known as Wilkie’s syndrome or Benign duodenal stasis, is a rare benign disease. It could threaten the life if the manifestation is severe and the treatment is inappropriate. In the patients with SMA syndrome, the third portion (transverse part) of the duodenum is compressed externally between the SMA and abdominal aorta (AA) leading to duodenal stasis and gastrointestinal obstruction. SMA syndrome may rarely combine with Nutcracker syndrome when left renal vein (LRV) was compressed between SMA and AA.

**Case presentation:**

A 32-year-old female patient presented with complaints of gradually severe bloating, epigastric pain, left flank ache, nausea and occasional vomiting of 1 month’s duration. The epigastric and left flank ache was aggravated when the patient was supine and relieved in a prone or left lateral decubitus. The abdominal bloating was associated with early satiety. The vomiting always started 40 min after meal. The patient gave a history of urine stone with drotaverine hydrochloride tablets treatment for two weeks before the gastrointestinal symptoms arising. The patient had no significant surgical history, but had a rapid weight loss of approximately 10 kg with a body mass index (BMI) from 21 kg/m^2^ to less than 18 kg/m^2^ over the last two months. An abdominal examination revealed upper abdominal tenderness and distention. The urine routine examination showed no significant abnormality. The findings of initial blood tests and other laboratory investigations were unremarkable.

**Conclusions:**

This case reports a female patient with SMA syndrome with Nutcracker syndrome predisposed by Antispasmodics. We highlight the importance of the combination therapy of long-term nutritional supporting and prokinetic agents. Rehabilitating practice after discharge is beneficial to reduce recurrence.

## Background

Superior mesenteric artery (SMA) syndrome, also known as Wilkie’s syndrome or Benign duodenal stasis, is a rare benign disease. It has reported that the prevalence of SMA syndrome was approximately 0.0024–0.3% [1–3]. Although SMA syndrome is benign, it could threaten the life if the manifestation is severe and the treatment is inappropriate [4]. In the patients with SMA syndrome, the third portion (transverse part) of the duodenum is compressed externally between the SMA and abdominal aorta (AA) leading to duodenal stasis and gastrointestinal obstruction [5]. The symptoms of SMA syndrome include acute or chronic episodic epigastric ache, bloating or vomiting, which associated with rapid growth in children and rapid weight loss in adults, particularly in young women [6, 7]. Once the compression of left renal vein (LRV) was just happened by SMA and AA, the SMA syndrome may rarely combine with Nutcracker phenomenon [8]. Here, we report a case of SMA syndrome coexist with Nutcracker syndrome in a 32-year-old female who presented to our gastroenterology department with a 1-month history of epigastric ache, bloating and vomiting after meal. In this report, we will further discuss the possible predisposing factors and other information for this case.

## Case presentation

A 32-year-old female patient presented to the gastroenterology department of the First Affiliated Hospital, Jinan University, Guangzhou, China, in 2017 with complaints of gradually severe bloating, epigastric and left flank ache, nausea and occasional vomiting of 1 month’s duration. The epigastric and left flank ache was aggravated when the patient was supine and relieved in a prone or left lateral decubitus. The abdominal bloating was associated with early satiety. The vomiting always began 40 min after meal. The patient provided a history of urine stone with oral drotaverine hydrochloride tablets treatment 40 mg three times a day (tid) for two weeks before the gastrointestinal symptoms arising. The patient had no significant surgical history, but had a rapid weight loss of approximately 10 kg with a body mass index from 21 kg/m^2^ to 18 kg/m^2^ over the last two months. An abdominal examination revealed upper abdominal tenderness and distention. The urine routine examination showed no significant abnormality (no hematuria and proteinuria). There were no remarkable abnormalities during the initial blood tests and other laboratory investigations.

**Fig. 1:**
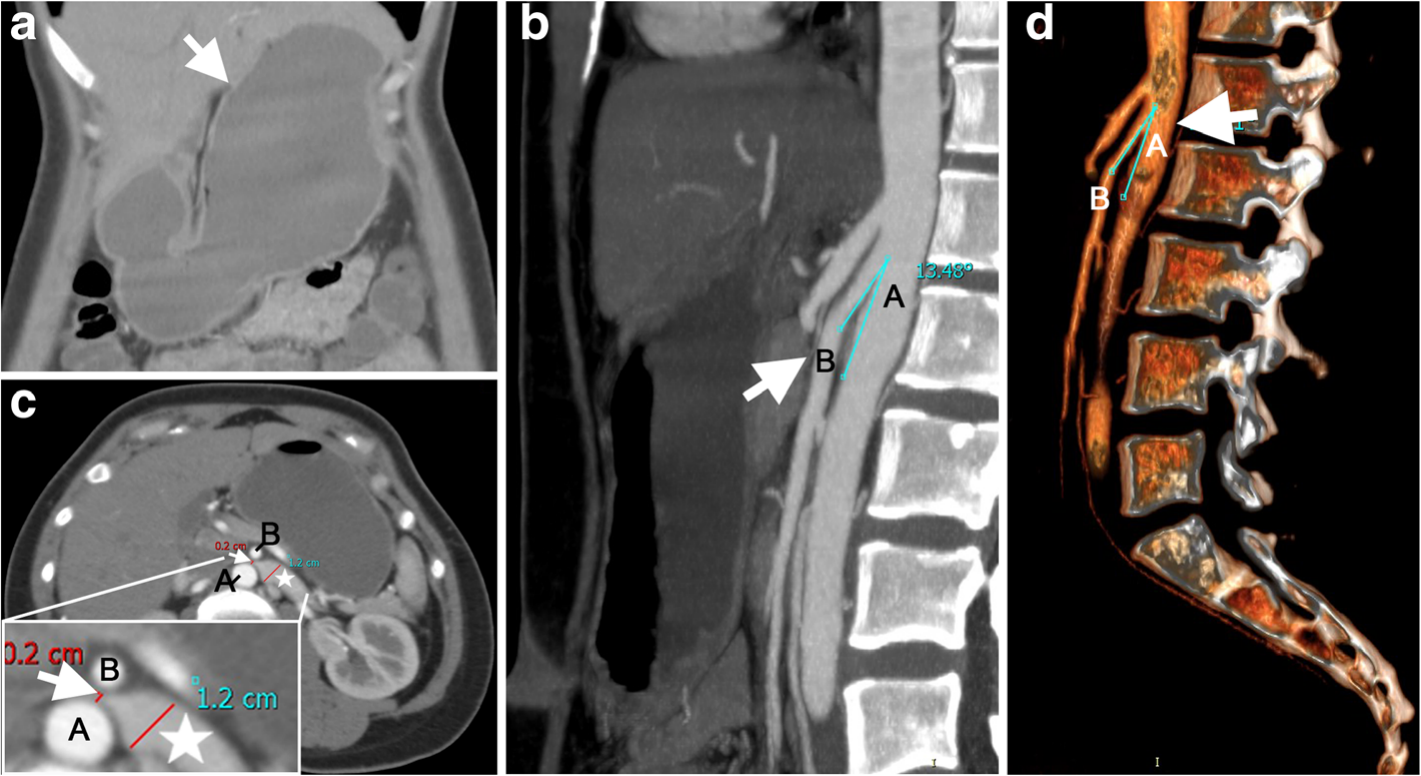
CT imaging showing SMA syndrome and Nutcracker Syndrome. (**a**) Coronal view of gastroduodenal dilation (arrow). (**b**) Sagittal view of aortomesenteric region. The angle between the AA “A” and the SMA “B” was less than 35° (measured at 13°) (**c**) The third portion of the duodenum was compressed by the decreased aortomesenteric angle in the transverse plane. The compressed LRV between the AA “A” and the SMA “B” was showed as arrow (bird beak sign). The dilation LRV was showed as star. The zoomed structures presented in the lower left. (**d**) Three-dimensional reconstruction of the AA “A” and the SMA “B”

**Fig. 2:**
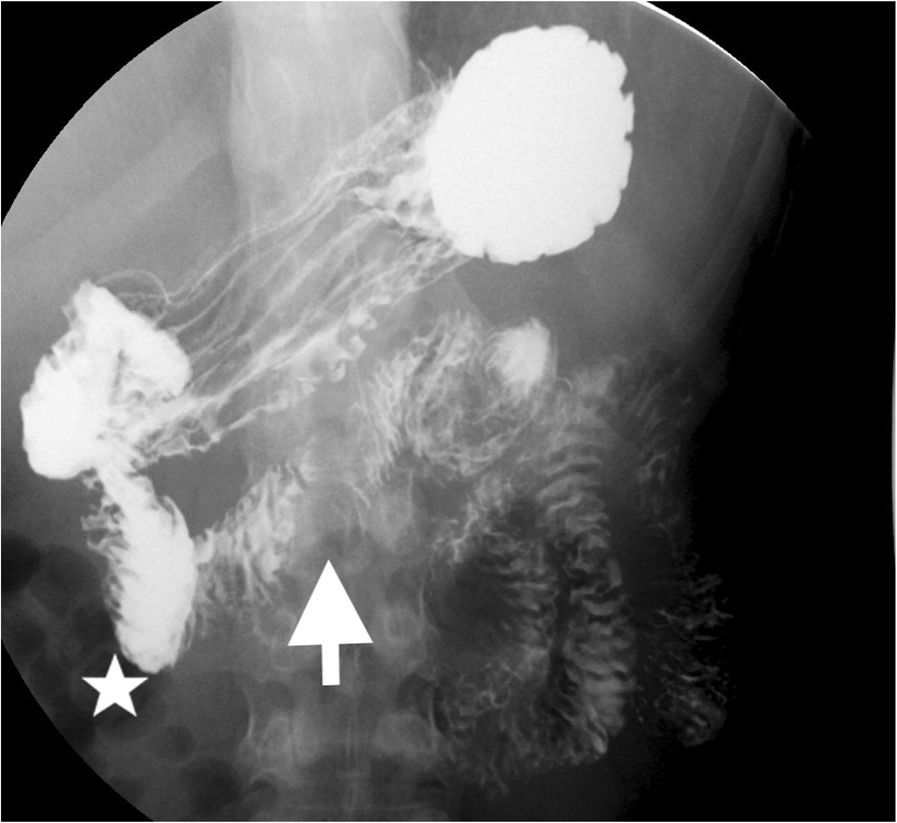
Upper gastrointestinal series/Upper gastrointestinal double-contrast radiograph. A vertical band of extrinsic compression (arrow) on the mid transverse part of duodenum compressed by SMA with proximal duodenal dilatation (star)

On performing a physical examination, her epigastric region was distended and tender to palpation. Contrast-enhanced abdominal computed tomography (CT) demonstrated gastroduodenal dilatation (Fig. 1a). There was narrowing of the third portion of the duodenum compressed by SMA and AA, with a decreased aortomesenteric distance of 3.7 mm and a narrower aortomesenteric angle of less than 15 degrees, which suggested a diagnosis of SMA syndrome (Fig. 1b, arrow). In addition, the LRV was compressed to 2 mm between SMA and AA (Fig. 1c, arrow), with a 12 mm dilatation in diameter (Fig. 1c, star), which formed a “bird beak sign” (Fig. 1c, arrow). Upper gastrointestinal double-contrast radiograph showed a vertical band of extrinsic compression (Fig. 2, arrow) on the mid transverse part of duodenum caused by SMA with proximal duodenal dilatation (Fig. 2, star). Gastroptosis was also observed by fluoroscopy. Colour Doppler indicated that the inside diameter of compressed stenosis of LRV was 1.4 mm on the left edge of AA, with 61 cm/sec of the maximum blood flow velocity. The inside diameter of proximal dilatation site was 6.1 mm, with 20 cm/sec of the maximum blood flow velocity. Additionally, the angle between SMA and AA was approximately 13 degrees, and bilateral iliac vein flow was slow. Therefore, a diagnosis of SMA syndrome and Nutcracker syndrome was confirmed.

Nasogastric tube was placed for decompression. Fluid resuscitation with parenteral and enteral nutritional support was managed conservatively to improve weight gain. Considering the neuromuscular and motility function could be impaired by the disease, pharmacotherapy (Mosapride citrate dispersible tablets 5 mg po tid) and physiotherapy (Functional gastrointestinal treatment apparatus) were treated to modulate the gastrointestinal motor function. Patient underwent a gastroduodenoscopy after the condition was relieved, revealing no intrinsic obstructions. Considering no significant abnormalities in renal function parameters and blood pressure, no special medical interventions were performed for the Nutcracker syndrome. One week later, the patient was discharged and subsequently received family nutrition support treatment for six months.

### Follow-up

**Fig. 3:**
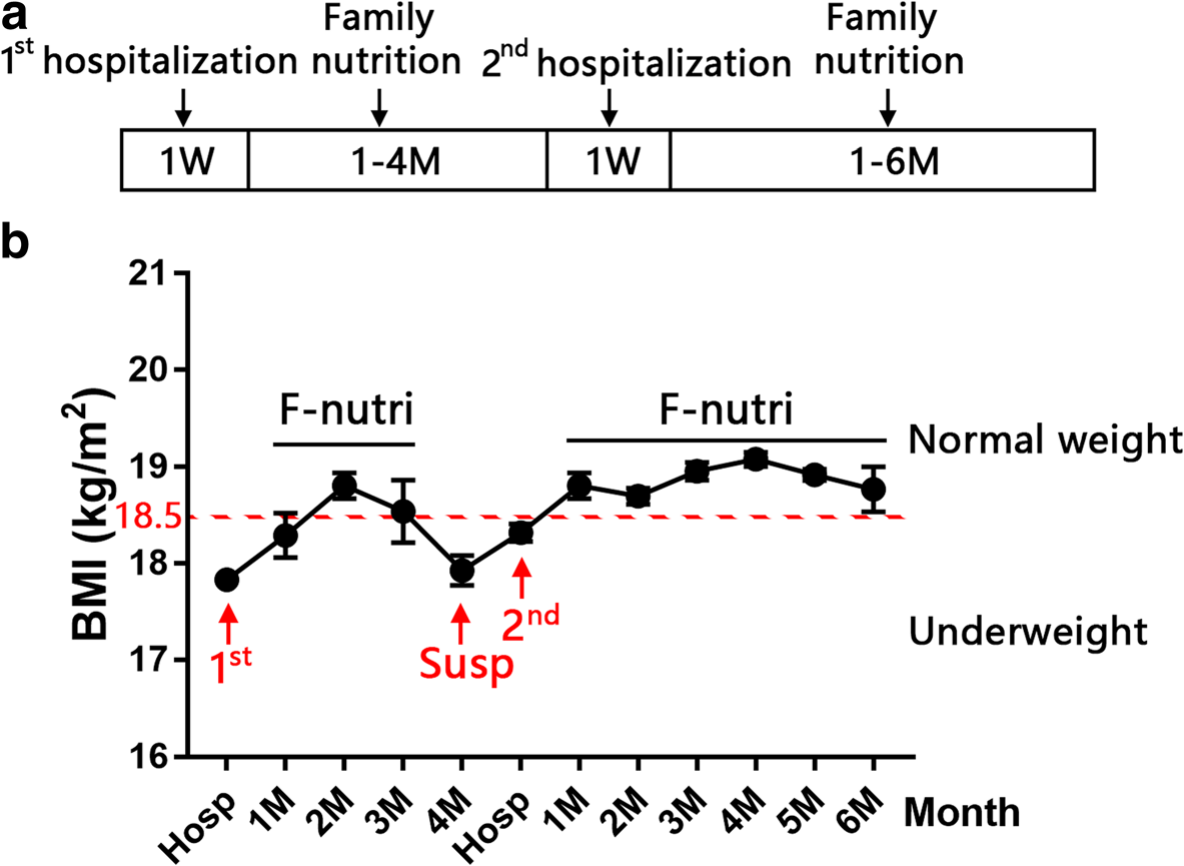
Follow-up BMI data collection. (**a**) The timeline of the disease course in this case report. (**b**) The follow-up BMI data collection. Hosp: hospitalization, F-nutri: family nutrition support, Susp: suspend

BMI data were collected each week during the family nutrition support treatment (Fig. 3a), and BMI values gradually increased (Fig. 3b). However, in the fourth month, she suspended the family nutrition support and began to work. BMI quickly reduced, and the symptoms recurred including bloating, epigastric pain, left flank ache and nausea. During her second hospitalization, an abdominal examination revealed upper abdominal distention, and the urine routine examination showed hematuria. Nasal jejunal tube was placed and Enteral nutrition was provided for one week. After weight gain and hematuria disappeared, the patient discharged. Subsequently, the patient received family nutrition support treatment for six months, and BMI values fluctuated within the normal range (18.8 ± 0.13 kg/m^2^ to 19.1 ± 0.74 kg/m^2^).

## Discussion

### Predisposing factors

The SMA syndrome is due to the lack of retroperitoneal fat and lymphatic tissue, which support a protection from the compression by SMA [9]. A number of predisposing factors leading to the abnormally narrow angle between the proximal SMA and AA have been described. Rapid growth in children and exaggerated weight loss in adults were the most common factors. Others include poor motility of the digestive tract, malabsorption, cachexia, visceroptosis, abdominal wall laxity, peritoneal adhesions, retroperitional tumors and catabolic states. The patient we reported had a lanky body build and a history of episodic epigastric pain and bloating for 25 years. The stressful career made her loss almost 10 kg of weight within two months, which was considered to be one of the predisposing factors leading to the acute symptoms. Two weeks before the gastrointestinal symptoms arising this time, she took antispasmodics (drotaverine hydrochloride tablets, 40 mg tid) for urine stone. However, antispasmodics may also cause smooth muscle relaxation in gastrointestinal tract. We supposed that spasmolytic drugs leaded to the disorder of the gastrointestinal motility, which aggravated the symptoms of duodenal obstruction and stomach retention. However, the causal relationship between spasmolytic drugs and SMA syndrome still need to be clarified through retrospective studies or prospective clinical trials. Therefore, all of the histories made us infer that multiple and complicated predisposing factors triggered the clinical symptoms arising based on her congenital anomalous anatomic characteristics.

### Diagnosis

#### SMA syndrome

Patients with the chronic, congenital SMA syndrome always have a lengthy and episodic history of abdominal complaints, such as epigastric bloating or anorexia. It is important to note that this condition is easily to be misdiagnosed as functional disorder or anxiety state, because the Gastrointestinal Endoscopy always display normal mucosa during the clinical diagnosis [10]. This female patient we reported had a history of episodic epigastric pain and bloating for 25 years, which was diagnosed as dyspepsia. Due to the intermittent exacerbating symptoms, she was suggested to fill in Hamilton Anxiety Scale once. Therefore, differential diagnosis is extremely important, since the misdiagnosis in this syndrome could be dangerous.

#### Nutcracker syndrome

The Nutcracker phenomenon may give rise to the Nutcracker syndrome in the presence of typical clinical manifestations with hematuria, proteinuria and left flank pain. This patient suffered from a left flank pain, without hematuria in the first hospitalization. Beside CT scan, the colour Doppler indicated the nutcracker phenomenon due to the compression of LRV and the abnormally small angle between SMA and AA. A transient hematuria occurred with a left flank pain during the second hospitalization, and disappeared after weight gain. Therefore, Nutcracker syndrome was diagnosed according to the imaging changes and clinical manifestations.

#### “Radiation-free” alternatives

The CT-scan was performed to screen the cause of abdominal pain. In order to confirm duodenal stasis and achieve a diagnosis of SMA syndrome, the upper gastrointestinal double-contrast radiograph was performed after then. However, alternative “radiation-free” imaging examination would be considered to avoid the radiation exposure, especially for the female patients of childbearing age. Ultrasonography (US) examination has been used in SMA syndrome diagnosis. It has been reported that abdominal US with power colour Doppler was performed for 3622 patients with dyspepsia and/or abdominal pain, revealing a significant reduction of AA [11]. SMA syndromes with Crohn’s disease underwent magnetic resonance enterography (MRenterography, MRE) drinking contrast medium in the prone position during the examination, which showed a decrease of aortomesenteric distance and an interruption of the third portion of duodenum [12].

### Management

#### Conservative treatment

Medical treatment may consist of nasogastric intubation for gastroduodenal decompression, reversal or removal of the precipitating factor and nutritional supplementation with hyperalimentation by jejunal feeding tube or peripherally inserted central catheter (PICC line). Prokinetic agents (such as Mosapride) or physiotherapy (such as functional gastrointestinal treatment apparatus) will be beneficial to modulate the gastrointestinal motor function. After the typical symptoms were relieved, small feeding and liquid diets would be available. Mobilization into the prone or left lateral decubitus position after eating should be practiced to relief duodenal decompression and improve gastric empty. The abdominal complaints will be improved after recovery of the lost weight.

#### Surgical treatment

Treatment of SMA syndrome initially involves conservative management. However, surgical intervention will be requited if medical treatment fails or the condition is severe [13]. The alternative operations mainly included open duodenojejunostomy and laparoscopic duodenojejunostomy. A prospective study in a single institution has been reported that SMA syndrome patients underwent duodenojejunostomy or performed a distal duodenum resection [1]. Laparoscopic duodenojejunostomy is considered to be feasible, safe, less morbid and effective for SMA syndrome compared with open surgery [14, 15]. Even for SMA syndrome combined with Nutcracker syndrome due to severe weight reduction, weight gain and SMA syndrome was corrected by laparoscopic duodenojejunostomy [16].

#### Rehabilitation

Besides nutritional supporting [17] and decubitus position, the rehabilitating practice after discharge is also important to reduce recurrence. We recommend the rehabilitating practice after discharge, such as appropriate swimming, which is beneficial to enhance the abdominal wall and reduce the frequency of recurrence, especially for female patient. However, retrospective study of more cases, even the prospective clinical trials are still needed to assess the effectivity.

## Conclusions

This case reports a female patient with SMA syndrome with Nutcracker syndrome predisposed by Antispasmodics. We highlight the importance of the combination therapy of long-term nutritional supporting and prokinetic agents. Rehabilitating practice after discharge is beneficial to reduce the frequency of recurrence.

## Abbreviations

AA: Abdominal aorta
BMI: Body mass index
CT: Contrast-enhanced abdominal computed tomography
LRV: Left renal vein
PICC: Peripherally inserted central catheter,
SMA: Superior mesenteric artery
